# Points from the Quarterly Report

**Published:** 1917-03-10

**Authors:** 


### THE LONDON HOSPITAL.

### Points from the Quarterly Report.

The Report presented to the Quarterly Court of Governors of the London Hospital, held on March 7, dealt with the arrangements made with the London County Council in connection with the treatment of venereal diseases. The scheme is in full working order, although no formal agreement has yet been completed with the London County Council.

Bacteriological examinations of blood, etc., are made for any local practitioner in the Home Counties, the County of London, and adjacent county boroughs. On notification, the necessary apparatus for collecting a specimen is sent, and a report on the result of the examination follows on the receipt of the specimen. Should the examination reveal a venereal disease free treatment is offered for the patient. Every care is taken to preserve secrecy, and the patient is known by a number and not by a name at the hospital. One book only, which is kept under lock and key, gives us the name and address of the persons whose numbers are on the register, and is used only for private communications between the doctor and the patient should it be necessary.

Should the patient prefer to be treated by his own doctor, the hospital supplies the necessary remedy free of charge to the doctor, should his name have been sent by the London County Council as "approved." This means that he has been through a course of instruction in the administration of the remedies.

In order that as many doctors as possible may become approved, free instruction is given in the hospital in every department of the work and at each of the sessions.

Increase in Charges of the Private Staff Nurses.

In view of the great drain on the nursing staff through the requirements of the War Office, the House Committee have felt it necessary to increase the weekly charge for supplying private nurses from two guineas to two and a-half guineas a week.

American and Lady Doctors.

As the War Office has had to call up so many house physicians and house surgeons, the Committee has obtained the services of six very highly qualified American doctors. These doctors have proved themselves quite invaluable to us, and we cannot be sufficiently grateful to Professor Foster Kennedy, of New York, who selected them, for the trouble he has taken in the matter.

The Cunard and the White Star Lines have generously assisted by allowing 25 per cent. off the passage money of doctors coming to England to give assistance to the hospitals in this time of need. Since the last Court six medical women have been engaged, and are giving efficient assistance.

Roll of Honour.

It has been decided that a Roll of Honour be put up at the main entrance, on which will be inscribed the names of members of the hospital staff, both medical and lay, who have been killed in the present war. Mr. Russell Howard, one of the members of the surgical staff, has most kindly offered to defray the cost.

Staff Investment in War Loan.

A scheme was drawn up by Mr. Douro Hoare, the Chairman of the Finance Committee, whereby members of the nursing staff, as well as the lay staff of the hospital, might subscribe to the War Loan. £4,450 has been so subscribed, of which £3,145 was from the nursing staff.

### Loan of Motor-car.

Owing to the serious difficulties experienced by the staff in attending the hospital at night for emergency operations, an advertisement was inserted in the papers asking for the loan of a car during the war. Eighteen or twenty gentlemen most generously put their cars at the service of the hospital.

The Committee accepted a Panhard car kindly lent by Mr. Emslie I. Horniman for the period of the war. The Committee are at present trying to secure a chauffeur.

### Coal Difficulties.

The Committee again express their indebtedness to Mr. Longstaffe, of Messrs. Sergeant, Longstaffe and Co., for the great personal trouble he has taken in keeping the hospital supplied with coal.

The War Office has informed the hospital that the Army Service Corps is willing to lend assistance in the delivery of coal should the danger of non-supply become acute.

### Beds and the War Office.

The War Office has again made application for beds for wounded men. Hitherto the Committee has been moved by consideration that it is most important that adequate help should be given in the East End to the civilian sick poor. It is true there is less sickness about at present, but both Bethnal Green and Mile End Infirmaries have been taken over by the War Office, and therefore the sick poor have less accommodation than usual in the East End. The Committee therefore have replied that while the "London," as ever, will be glad to render assistance to the War Office in case of urgent need and when military beds are full, the Committee feels that it must be the Government who shall take the responsibility of deciding that civilian beds are to be closed in order that they may be occupied by the military. The hospital has in the wards 100 officers now, and has offered to take another 100 men; this can be done without seriously interfering with the civilian beds at all. There is also a pledge to supply 250 beds to the Admiralty, if necessary.

In this connection a grant was received from the Army authorities of £508 18s. 5d. for the cost of equipment of the officers' wards.

